# Low Osteoporosis Treatment Initiation Rate in Women after Distal Forearm or Proximal Humerus Fracture: A Healthcare Database Nested Cohort Study

**DOI:** 10.1371/journal.pone.0143842

**Published:** 2015-12-02

**Authors:** Marie Viprey, Pascal Caillet, Guillaume Canat, Susan Jaglal, Julie Haesebaert, Roland Chapurlat, Anne-Marie Schott

**Affiliations:** 1 Pôle Information Médicale, Evaluation, Recherche, Hospices Civils de Lyon, Lyon, France; 2 Faculté de Médecine Lyon Est, Université Claude Bernard Lyon 1, Lyon, France; 3 Département d'épidémiologie, hygiène hospitalière et santé publique, Centre Hospitalier Universitaire d’Amiens, Amiens, France; 4 INSERM U1033, Lyon, France; 5 Département de l’Information Médicale, Groupe Hospitalier Sud Réunion, Centre Hospitalier Universitaire de la Réunion, Saint Pierre, France; 6 Department of Physical Therapy, Toronto Rehabilitation Institute-UHN Chair, University of Toronto, Toronto, Canada; 7 Service de Rhumatologie et de Pathologie Osseuse, Groupement Hospitalier Edouard Herriot, Hospices Civils de Lyon, Lyon, France; Garvan Institute of Medical Research, AUSTRALIA

## Abstract

Treatment initiation rates following fragility fractures have often been reported to be low and in recent years numerous programs have been implemented worldwide to increase them. This study aimed at describing osteoporosis (OP) treatment initiation in a representative sample of women who were hospitalized for a distal forearm fracture (DFF) or proximal humerus fracture (PHF) in 2009–2011 in France. The data source was a nationwide sample of 600,000 individuals, extracted from the French National Insurance Healthcare System database. All women aged 50 years and older who were hospitalized for a DFF or PHF between 2009 and 2011 and who had not received any OP treatment in the preceding 12 months were included in a retrospective cohort study. OP treatments initiated during the year following the fracture were analyzed. From 2009 to 2011, 729 women were hospitalized for a DFF or a PHF and 284 were on OP treatment at the time of the fracture occurrence. Among the 445 women who had no prevalent OP treatment, 131 (29.4%) received supplementation treatment only (vitamin D and/or calcium) and 42 (9.4%) received a pharmacologic OP treatment in the year following their fracture. Pharmacological OP treatments included bisphosphonates (n = 21), strontium ranelate (n = 14), hormone replacement therapy (n = 4), or raloxifene (n = 3). General practitioners prescribed 75% of initial OP treatments. Despite the guidelines published in 2006 and the numerous initiatives to promote post-fracture OP treatment, OP treatment initiation rate in women who were hospitalized for a fragility fracture remained low in 2009–2011 in France.

## Introduction

Osteoporosis (OP) is characterized by reduced bone mass and disruption of bone architecture, resulting in increased bone fragility and increased fracture risk [1]. Fragility fractures like distal forearm fractures (DFF or wrist fracture) have been described as an early sign of OP [2–4]. The incidence of DFF increases with age, which makes DFF the most frequent fracture in postmenopausal women [5–7]. Proximal humerus fractures (PHF) are the third most frequent non-vertebral fractures in patients over 65 following wrist and femoral neck fractures [7,8]. It has been shown that a personal history of wrist fracture is a major independent risk factor for a future fragility fracture, and particularly femoral neck fracture [9,10]. Despite the demonstrated efficacy of OP treatment in the secondary prevention of osteoporotic fractures [11], several studies conducted in the United States, Canada, Europe and Australia have indicated that although treatment rates for osteoporosis were improving, they still appeared suboptimal, especially regarding post fracture care [12–19]. The study of Andrade et al. [20] conducted in USA in 2003 showed that only 24% of women 60 years of age or older who sustained a fragility fracture underwent initiation of OP treatment with bisphosphonates (BP), hormonal replacement therapy (HRT), or calcitonin in the year following the fracture.

In the early 2000’s, numerous post-fracture care programs were implemented to improve OP investigation and prevention of further fragility fracture worldwide [21,22]. Parallel to these, in France, institutional guidelines were issued in 2006 by the French national authority for health (*HAS*) [23]. Since these initiatives to promote post-fracture treatments, no study assessing the current trends in OP treatment initiation after the occurrence of fragility fracture has been conducted.

The main objective of this observational study was to evaluate the OP treatment initiation rate after a hospitalization for distal forearm or proximal humerus fracture in 2009–2011 in a French population of women without OP treatment at the time of fracture. The secondary objective was to describe OP treatments when initiated.

## Methods

A retrospective cohort study was carried out using a nationwide representative sample of the French National Insurance Healthcare System database.

### Data source

We used data provided by the “*Echantillon Généraliste des Bénéficiaires*” (*EGB*), a permanent representative sample of the general population of subjects affiliated with the National French health insurance system. The EGB currently includes more than 600,000 patients [24], and contains reimbursement data for hospitalizations, drugs, and tests ordered by physicians.

Our national dataset, in accordance to the laws that regulate hospital database in France, namely articles L. 6113–7 and L. 6113–8 of the Public Health Code, was anonymized by hospitals, ATIH (French agency of hospital information) and the French Insurance Healthcare Fund. All legal conditions for epidemiological surveys were respected, and the French national commission governing the application of data privacy laws (the “*Commission Nationale Informatique et Libertés*”) issued approval for the project. Since the study was strictly observational and used anonymous data, in accordance to the laws that regulate “non-interventional clinical research” in France, namely articles L.1121-1 and R.1121-2 of the Public Health Code, did not require the written informed consent from the participants or the authorization from any other ethics committee to conduct this survey.

### Study sample

The source population was composed of all women aged 50 years or older in the EGB database who were hospitalized for a DFF or a PHF between January 1, 2009 and December 31, 2011. Fractures were identified by the use of ICD-10 codes (S52.5-S52.6 for DFF and S42.2 for PHF) as a primary diagnosis or a procedural code of orthopaedic reduction of fracture of the distal end of one bone or both bones of the forearm, or osteosynthesis of fracture of the distal end of a bone of the forearm. The index date for the analysis was the date of the fracture. Each patient was followed for one year starting from the index date. Patients were not included if they received OP drugs during the 12-months period before the fracture; sustained a DFF or a PHF during the year before the index date; changed health insurance during the study period; had a history of primary or secondary neoplasms of bone and articular cartilage (C79.5 as primary diagnosis), hypercortisolism (E24 as primary diagnosis) or myeloma (C90.0 as primary diagnosis) in the previous year or 6 months after the index date; received treatment with corticosteroids more than 3 months during the 12-months period before the fracture; or had an intervention on parathyroid or thyroid glands 3 months before the fracture or the 6 months after (E21, E21.4, E21.5, E05, E06 as a primary diagnosis or a procedural code of medical or surgical procedure on parathyroid glands or on thyroid of the *CCAM*).

To describe the cohort, we anonymously extracted the following demographic data from the database: date of birth, healthcare setting (public teaching hospital center, public non-teaching hospital center or private hospital center), “*Couverture maladie universelle*” (*CMU*) status which identifies patients with low income, number of reimbursements for corticosteroids, and number of reimbursements for BMD tests, and “*Affection de longue durée*” (*ALD*) status. The *ALD* status identifies patients with a major chronic disease coded according to the International Classification of Disease, 10th version (ICD-10), as reported by their general practitioner and approved by a physician employed by the National Healthcare Insurance. The French Healthcare Insurance Fund has already been described elsewhere [25].

### Osteoporosis treatment initiation

OP treatment initiation was defined as a reimbursement for bisphosphonates (i.e. etidronate, alendronate, ibandronate, risedronate, zoledronate), hormonal replacement therapy (HRT), raloxifene, strontium ranelate, calcium, vitamin D, or teriparatide, with no prior reimbursement for one of these drugs during the year preceding the index date. OP drugs were identified by their “*Code Identifiant de Présentation” (CIP)*, which is a unique identification number for each drug assigned by the French health authorities when marketed. The primary outcome of this study was OP treatment initiation rate. We also examined characteristics of patients and prescribers who initiated OP treatment and evaluated as a secondary outcome the time between fracture and OP treatment initiation. The OP treatment initiation rate was calculated as the proportion of patients hospitalized for a DFF or PHF who had OP treatment initiated in the following 12 months. For each OP drug, the following variables were extracted: *CIP*, dates of prescription, dates of dispensation and prescriber’s specialty. For public hospital practitioners, specialty was often missing thus they became a specific category in the analysis. Additionally, we analysed BMD tests prescribed after the fracture.

### Statistical analysis

Results are presented as numbers and percentages of subjects for categorical variables and as means and standards deviations for continuous variables (or median and interquartile range for non-normally distributed variables). To describe the study population compared to the source population, the denominator used to measure the percentages was the total number of fractured patients, regardless of prevalent OP treatment. To describe incident treatments in the study population, the denominator used to compute incidence proportions was the number of fractured patients without prevalent OP treatment. OP treatment initiation proportions were also analysed according to the specialty of the prescribing physician, patient’s age, context of care, *CMU* and *ALD* status, presence of a reimbursement for a BMD test within 12 months after the fracture, and presence of long term corticosteroid therapy within 12 months after the fracture. Between-group comparisons were performed with χ² tests for categorical variables. The threshold for statistical significance retained for the analyses was p = 0.05. Data were analysed centrally using SAS® Enterprise Guide Software V.4.3 (SAS Institute Inc., Cary, NC, USA).

## Results

**Fig 1:**
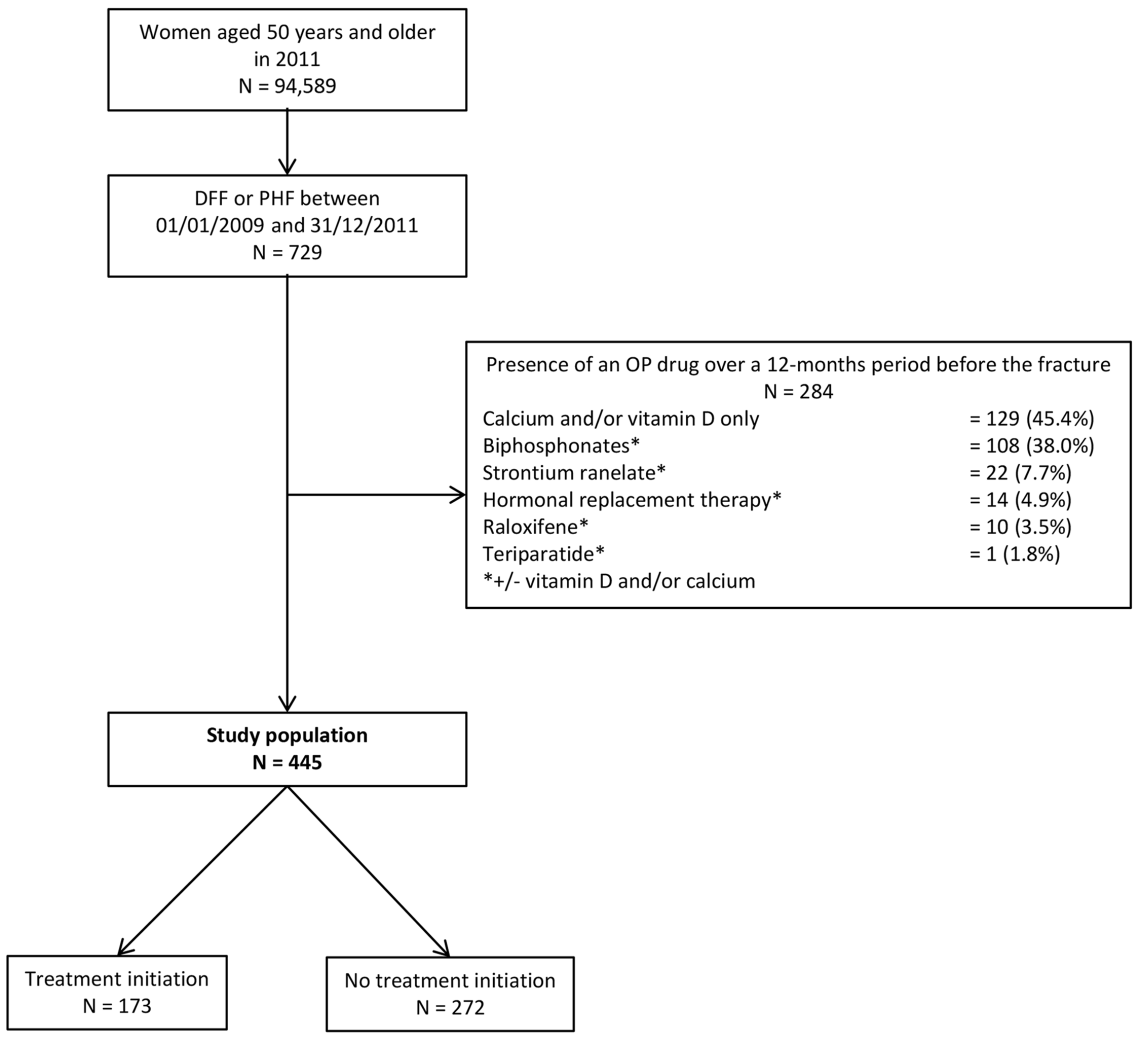
Flowchart of the study.

Among the 729 women aged 50 years or older who were hospitalized for a DFF or a PHF over the study period, 284 (39.0%) received OP drugs in the 12-month period before their fracture and thus, were not included in the study population (Fig 1). These 39.0% were distributed as follows: 21.3% (155 women) who received a pharmacologic OP treatment including bisphosphonates, strontium ranelate, HRT, raloxifene or teriparatide and 17.7% (129 women) who received supplementation treatment only (vitamin D and/or calcium).

The remaining 445 women who were hospitalized for a DFF or a PHF from January 1, 2009 to December 31, 2011 without prevalent OP treatment constituted the study population. Among those, 173 women (38.9%) had OP treatment initiation after the fracture.

Characteristics of initiated OP treatments initiated during the year following the fracture in the study population are described in Table 1. Among the 173 newly initiated OP treatments, 131 (29.5% of the study population) were supplementation only with vitamin D and/or calcium and 42 (9.4% of the study population) were pharmacologic OP treatments including bisphosphonates, strontium ranelate, HRT or raloxifene. General practitioners (GPs) prescribed three quarters (n = 130) of initiated OP treatments, whereas rheumatologists and gynaecologists represented less than 10% (n = 17). The median time elapsed between fracture and OP treatment initiation was 96 days, with an interquartile range between 49 and 184 days. Among the 173 women who initiated an OP treatment in the year following their fracture, only 48 had a BMD test in the year following the fracture.

**Table 1:** Description of initiated osteoporosis treatments (N = 173).

Characteristics	
Median time elapsed between fracture and OP treatment initiation (in days; IQR)	**96 (49–184)**
Type of osteoporosis treatment initiated	** **
Supplementation treatment only (n, %)	**131 (75.7)**
Vitamin D alone	89 (51.4)
Calcium and vitamin D	40 (23.1)
Calcium alone	2 (1.2)
Pharmacological osteoporosis treatment (n, %)	**42 (24.3)**
Bisphosphonates^1^	21 (12.1)
Strontium ranelate^1^	14 (8.1)
Hormonal replacement therapy^1^	4 (2.3)
Raloxifene^1^	3 (1.7)
Physician specialty (n, %)	** **
General practitioner	130 (75.1)
Rheumatologist	9 (5.2)
Gynecologist	8 (4.6)
Other private practitioner	3 (1.7)
Public hospital practitioner	23 (13.3)

^**1**^ ± vitamin D and/or calcium.

Differences in characteristics between the group of patients with initiation of treatment and the group of patients without treatment initiation were not statistically significant (Table 2), except for the proportion of patients who had a BMD test in the year following the fracture, which was greater in the group of “treated patients” (27.7% vs 4.8%; p<0.001). Overall, 61 (13.7%) of the 445 women hospitalized for a DFF or a PHF during the study period had a BMD test in the year following the fracture. Among these 61 women, 48 (78.7%) initiated an OP treatment, including supplementation treatments (n = 27) and pharmacologic OP treatments (n = 21).

**Table 2:** Characteristics of patients who were hospitalized for a proximal humerus fracture or a distal forearm fracture and had no prevalent osteoporosis treatment, by treatment group (N = 445).

**Characteristics**	**Patients with treatment initiation (N = 173)**	**Patients without treatment initiation (N = 272)**
Mean age (years) ± SD	70.3 (12.4)	68.1 (12.8)
**Healthcare setting**		
Public teaching hospital center	17 (9.8)	19 (7.0)
Public non-teaching hospital center	63 (36.4)	79 (29.9)
Private hospital center	93 (53.7)	174 (63.9)
*ALD* status^1^	22 (12.7)	27 (9.9)
*CMU* status^2^	7 (4.0)	21 (7.7)
Long term corticotherapy^3^	4 (2.0)	2 (0.7)
BMD test within 12 months after the fracture	48 (27.7)*	13 (4.8)*

Data are means ± SD or number of patients (% of total)

^1^ The *ALD* status identifies patients with a major chronic disease coded according to the International Classification of Disease, 10th version classification system (ICD-10), as declared by their general practitioner and approved by a physician employed by the National Healthcare Insurance

^2^ The *CMU* status identifies patients with low income

^3^ Long term corticotherapy defined as at least six reimbursements for oral or injectable corticosteroids in the year preceding the fracture

* p<0.001 with χ² test.

## Discussion

Among the 729 women aged 50 years or older who were hospitalized for a DFF or a PHF over the study period, 155 (21.3%) had a prevalent pharmacological OP treatment. Among the 445 women who had no treatment at the time of the fracture and constituted the study population, 131 received supplementation treatment only (vitamin D and/or calcium), 42 received a pharmacologic OP treatment, and only 61 had a BMD test in the year following their fracture. Despite guidelines and numerous initiatives to promote post-fracture OP treatment in the early 2000’s, both prescription of BMD and prescription of OP treatment in women who were hospitalized for a distal forearm or proximal humerus fracture remained low in 2009–2011 in France.

In the review of Elliot-Gibson V et al. [12], which included 37 studies of post-fracture OP investigation and treatment published between 1994 and 2003, treatment rates for OP in patients following a fragility fracture were relatively low and very different across studies. Rates for treatment initiation of vitamin D and calcium ranged from 8% to 62% and for bisphosphonates from 0.5% to 38%. In 2000, Freedman et al. [26] showed in a similar study to ours conducted on 1162 women in the USA that only 16.8% of women 55 years or older who sustained a distal radial fracture had OP treatment initiated with estrogen, bisphosphonates, or calcitonin (14.6%, 2.5% and 1.2% respectively, expositions not being exclusive). This was before the publication of WHI study [27] and current patterns would probably be different in the period 2009–2012. More recently in UK in 2007, Talbot JC et al. [28] showed that among 175 patients who suffered a distal radius fracture, 25.1% had OP treatment initiation. Treatments were based on calcium (22.2%), vitamin D (20.0%) or bisphosphonates (6.9%), not being exclusive of each other. We observed a higher initiation rate (38.9%), but as the authors did not exclude patients with OP treatment at the time of fracture, our results may not be comparable.

The OP treatment initiation rate may be dependent on fracture site. In the Global Longitudinal study of Osteoporosis in Women (GLOW) published in 2013 [29], 42% of the women aged 65 years or older with a history of hip or spine fracture recruited in Northern Europe were taking an OP treatment (bisphosphonates, raloxifene, teriparatide, tibolone, calcitonin, strontium ranelate, or HRT) at the time of inclusion, which is greater than the rate we observed regarding upper limb fractures. As the authors stated, major fractures like hip or spine fractures represent more severe health events, thus they may be more likely to attract attention of the treating physician and to trigger an OP treatment.

In 2012, Leslie et al. [30] published a historical cohort study (April 1, 1996 to March 31, 2008) of Canadian men and women aged ≥50 years who had experienced a non-traumatic fracture. The proportion of individuals receiving BMD testing or dispensation of an osteoporosis medication (bisphosphonate, calcitonin, selective estrogen receptor modulators, or HRT) in the 12 months following the fracture varied by fracture site: hip 15.1%, spine 37.5%, humerus 15.8%, forearm 18.9%. For individuals with major osteoporotic fracture (hip, spine, humerus, or forearm) who were not on treatment at the time of fractures, the proportion receiving post-fracture osteoporosis medication was 6.1% in 1996/1997, increasing to a maximum of 14.7% in 2003/2004 and declining to 8.3% in 2007/2008 (p-for-trend<0.001). This decline was probably due to the decrease if HRT after 2004 and tis study shows that OP treatments patterns vary not only across types of fractures but also across time periods.

We showed that the most frequent pharmacologic OP treatment initiated after a DFF or a PHF, other than HRT, were bisphosphonates (4.7%), followed by strontium ranelate (3.1%), and then raloxifene (0.7%). In a previous retrospective cohort study with all women living in Rhône-Alpes area and who underwent a first BMD test without prior OP treatment between 2006 and 2009, we found that 27% initiated an OP treatment (bisphosphonates, raloxifene, teriparatide, strontium ranelate, or estrogen) within 4 months, mostly with bisphosphonates (16.6%), less frequently with strontium ranelate (5.8%) or raloxifene (3.3%) [31]. We conducted another study in the Rhône-Alpes area in 2010 [32] and found that among 4,415 women over 50 years with a first claim for OP treatment, bisphosphonates were prescribed in 60.4% of the cases, strontium ranelate in 27.5% and raloxifene in 12.1%. This consistency in our results could be expected as the samples used for both previous studies were drawn in a similar timeframe. However, our current sample was drawn from a nationwide representative database, which increases the potential generalization of the previous results to the whole French population. Regarding prescriber specialty, our results are consistent with those from a previous study conducted in the Rhône-Alpes area which reported that, among women with ongoing OP treatment in 2006, most were prescribed by a GP (79.0%), fewer by a rheumatologist (11.6%) or a gynecologist (7.1%) [33].

The main strength of this study is that it was based on recent data, extracted from a representative sample of the exhaustive reimbursement database [24], which validity and usefulness in pharmaco-epidemiologic studies has been previously studied and validated [34,35]. Analysis of large medico-administrative databases offers advantages of completeness of patients and unbiased study sample. However they present several limitations which should be acknowledged. Firstly, reimbursement data were used as proxies for drug intake as in most pharmaco-epidemiologic studies conducted with medico-administrative databases. It could lead to an overestimation of compliance. However, Noize et al. [36] have found that agreement between reimbursement data contained in the French Insurance Healthcare Fund and the self-reported drug use at interview of patients was good (Kappa for drug treatments for bone disease = 0.73[0.67–0.79]). Another limitation is the impossibility of distinguishing between patients who have been supplied with a prescription but did not refill the prescription and no prescription from physician. Finally, we do not have information on patients who were not hospitalized but treated at the emergency department, therefore underestimating the real burden of these fractures. In a French study published in 2012, only 43% of the patients who sustained a PHF managed in an emergency department were hospitalized [37]. Our sample represents a subset of the more severe cases, requiring surgical treatments. Thus, we would expect the under diagnosis and under treatment of osteoporosis to be even more important in the overall population with less severe fractures.

Despite the guidelines published in 2006 and the numerous initiatives to promote post-fracture OP treatment, OP treatment initiation rate in women who were hospitalized for a DFF or a PHF without OP treatment at the time of the fracture remained low in 2009–2011 in France. Although these fragility fractures should alert and lead to diagnostic tests and treatments to prevent the risk of future osteoporotic fracture, they are still undertreated in most western countries.
